# Comparison of Two Commercial PCR Methods for Methicillin-Resistant *Staphylococcus aureus* (MRSA) Screening in a Tertiary Care Hospital

**DOI:** 10.1371/journal.pone.0043935

**Published:** 2012-09-19

**Authors:** Aylin Aydiner, Jessica Lüsebrink, Verena Schildgen, Ingo Winterfeld, Oliver Knüver, Katja Schwarz, Sabine Messler, Oliver Schildgen, Frauke Mattner

**Affiliations:** 1 Institut für Pathologie, Kliniken der Stadt Köln gGmbH, Köln, Germany; 2 Institut für Hygiene, Kliniken der Stadt Köln gGmbH, Köln, Germany; Rockefeller University, United States of America

## Abstract

Nose/throat-swabs from 1049 patients were screened for MRSA using CHROMagar MRSA, LightCycler Advanced MRSA, and Detect-Ready MRSA. Results were compared to the CHROMagar MRSA results, which was set as reference system. MRSA was detected in 3.05% of the patients with CHROMagar MRSA. LightCycler MRSA Advanced showed a higher clinical sensitivity (84.38%) than Detect-Ready MRSA (57.69%).The negative predictive values were high for both tests (>98%). The specificity and the positive predictive value were higher for the Detect-Ready MRSA test than for the LightCycler MRSA test (99.59% and 78.95% versus 98.52% and 64.29%). For routine screening LightCycler MRSA Advanced proved to be more efficient in our clinical setting as the clinical sensitivity was much higher than the sensitivity of Detect-Ready MRSA. CHROMagar MRSA detected more MRSA positive samples than both PCR methods, leading to the conclusion that the combination of PCR with cultural screening is still the most reliable way for the detection of MRSA. LightCycler MRSA Advanced was faster and needed less hands-on time. The advantage of Detect-Ready MRSA was the additional identification of methicillin-sensitive *S.aureus* (here in 34.63% of the samples), an information which can be possibly used for reducing the risk of postoperative infections in surgical patients in future.

## Introduction

Nosocomial infections caused by methicillin-resistant *Staphylococcus aureus* (MRSA) have been shown to be associated with severe clinical and economical outcomes [1]–[12] Whereas high MRSA prevalences were reported from countries like the USA, Taiwan, Japan, or the southern European countries (e.g. up to 80% of methicillin-resistant S. aureus of S. aureus in blood-cultures) the prevalences in the Netherlands or Scandinavia were low (<1–5%) [6], [7], [8], [9], [10]. These low prevalences appear to be the result of a more effective MRSA management [9], [10], consisting in MRSA screening at patient admission, isolating colonized patients in single-rooms or cohorting them, decolonization of MRSA-positive patients, and strict staff hand hygiene [13], [14].

However, screening methodology is crucial for detecting MRSA and no method is tested the most sensitive, specific or cost effective, so far. Inexpensive culture methods have the disadvantage of a swab-to-result time of up to 48 hours and different commercially available media show even differing accuracy values [35].

More expensive molecular-based test systems show more interesting turn-over-times but only few studies estimating accuracy values in a routine diagnostic setting are available so far [36]. Data of the Detect-Ready® MRSA Kit concerning these questions are still lacking.

In our study we compared the Detect-Ready® MRSA Kit (MDI, Kent, UK) with the standard methods used in our laboratory, the LightCycler® MRSA Advanced Test (Roche, Mannheim, Germany) and the CHROMagar MRSA II (BD, Heidelberg, Germany). Our screening comprised all patients at admission to our emergency department or two intensive care units in accordance with our routine MRSA screening management.

## Materials and Methods

### Settings and specimen collection

The study was conducted at the Kliniken der Stadt Köln, a 1500 bed tertiary care facility and university affiliated teaching hospital in the City of Cologne, Germany. During a four month period from August to November 2011 all patients admitted to two intensive-care units or the emergency department of the hospital were screened for MRSA and included into the study, leading to 1049 patients included. Specimens were collected by the nursing staff using double-headed swabs with amies gel (Copan, Italy). In each patient a combined swab was taken from the throat and both nares, the most important sites for MRSA colonization [15], [16], [17], [18]. Technically, the throat was swabbed rotating against the mucosa using a double-headed swab which was then used for swabbing both nares in the same way. The aim of the study was to evaluate two diagnostic assays for potential routine use, thus no ethical vote was required. The LightCycler® Advanced MRSA Assay (Roche) was performed as the routine diagnostic assay in our laboratory, whilst the Detect-Ready® Assay (Molecular Detection Inc) was performed in addition during the study period. Therefore verbal informed consent was sufficient. All procedures were performed according to the declaration of Helsinki in its present form.

### Laboratory processing and culture methods

**Figure 1:**
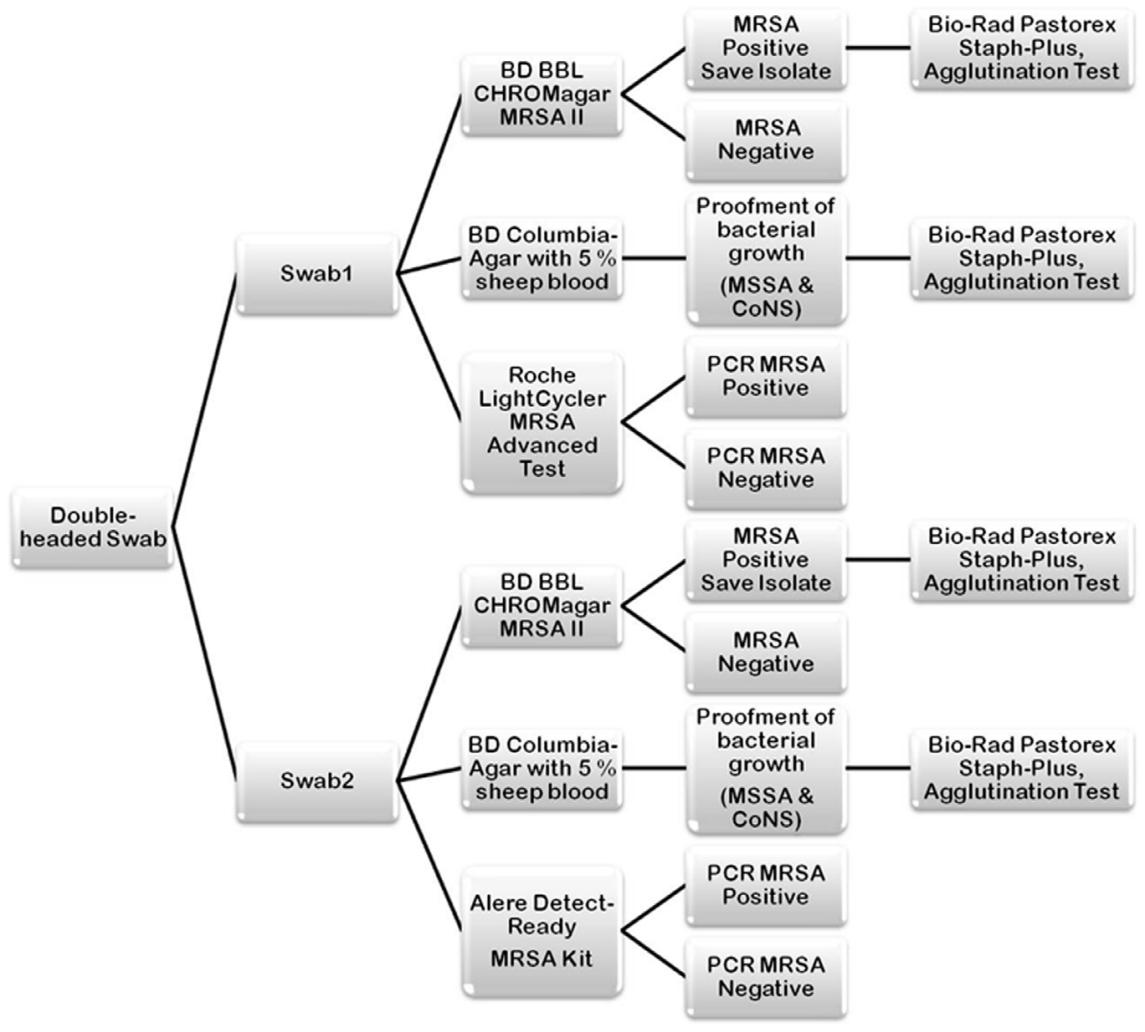
Clinical experimental design. Nasal/throat specimens were tested by Detect-Ready® MRSA Kit and by the LightCycler® MRSA Advanced Test. For testing, the double-headed swab was separated. One swab head was processed for directly plated culture on BD BBL™ CHROMagar™ MRSA Medium II, BD Columbia Agar with 5% sheep blood plates, and the LightCycler® MRSA Advanced Test. The other swab was used for the assay with Detect-Ready® MRSA Kit and directly plated culture on BD BBL™ CHROMagar™ MRSA Medium II, and BD Columbia Agar with 5% sheep blood plates. MRSA positive colonies onto the selective chromogenic agar were confirmed to be *S. aureus* using an agglutination test.

The BBL CHROMagar MRSA II (BD, Heidelberg, Germany) was used as screening agar for MRSA [19]. Additionally, Columbia-Agar with 5% sheep blood (BD) was used as control for bacterial growth and for identification of methicillin-sensitive *S. aureus* (MSSA) (Figure 1). In the laboratory both swabs of the double-headed swabs were firstly separated and each part was used for inoculating one MRSA selective agar and one Columbia agar consecutively. The two parts of the swab were then used for DNA-extraction for the one or the other PCR method, one for the LightCycler® MRSA Advanced Test, one for the Detect-Ready® MRSA Kit. The culture plates were incubated at 36°C and evaluated after 24 and 48 hours. Mauve-coloured colonies onto the selective chromogenic agar were confirmed to be *S. aureus* using an agglutination test for the simultaneous detection of the fibrinogen affinity antigen (clumping factor), protein A, and the capsular polysaccharides of *S. aureus* (Pastorex Staph-Plus, Bio-Rad, Munich, Germany). In case of agglutination the culture was sent to the routine diagnostics microbiology laboratory serving the Kliniken der Stadt Köln for species identification and antibiotic-resistance testing via the Vitek2 system (bioMerieux).

In case of the absence of typical (mauve coloured) colonies on the chromogenic medium and colonies suspected to be *S.aureus* on the Columbia agar, these colonies were analysed in the same way.

### PCR methods

The LightCycler® MRSA Advanced Test targets the integration site of the SCC*mec* cassette into the *S. aureus* chromosome (SCC*mec*:*orfX* junction). The Detect-Ready® MRSA Panel Kit simultaneously targets three MRSA DNA regions, namely the *mecA* gene, the *nuc* gene and SCC*mec*:*orfX* junction. In this way the assay is able to identify MRSA, MSSA and Coagulase negative staphylococci (CoNS) with the help of integrated software considering the different amplification rates. CoNS are assumed if the *mecA* gene is detected whereas the *nuc* gene and the SCC*mec*:*orfX* junction remain undetectable. Both, the LightCycler® MRSA Advanced Test and the Detect-Ready MRSA Panel Kit (Rotor-Gene compatible version, MDI, Kent, UK) were performed according to the manufacturer's instructions. DNA was extracted using the LightCycler Advanced Lysis Kit (Roche) for the LightCycler MRSA Advanced Test and the Detect-Ready MRSA Swab Lysis Kit (MDI) for the Detect-Ready MRSA Panel Kit according to the manufacturer's instructions.

Each PCR method contains an Internal Control (IC) to exclude or detect PCR inhibition and to monitor reagent integrity. The IC must be detected positive in all MRSA negative specimens otherwise the specimen is automatically set as “invalid” by the program software.

### Result definitions

A true positive (TP) result was defined as positive result in the PCR and the corresponding culture. A true negative (TN) result was defined as a negative result in the PCR and the corresponding culture. A sample positive in the PCR and negative in the corresponding culture was defined as false positive (FP), a sample negative in the PCR and positive in the corresponding culture was defined as false negative (FN).

### Strain typing

Cultured MRSA isolates from samples with negative PCR results were reanalyzed by PCR. Isolates with repeated negative result were genotyped with Identibac StaphyType (Clondiag, Jena, Germany). The analysis was conducted by Alere Technologies GmbH (Jena, Germany). In addition the SCC*mec* type was determined according to the method of Boye *et al.* [20].

### Data analysis

The results of the LightCycler® MRSA Advanced test and the Detect-Ready® MRSA test were compared to the corresponding CHROMagar MRSA plates. The clinical sensitivity, specificity, and the negative and positive predictive values (NPV and PPV) were calculated for each PCR method. Samples with invalid PCR results were excluded from the calculations.

## Results

### Patient profiles and culture results

Of the 1049 patients, 499 (47.57%) were female and 550 (52.43%) were male. Overall, 214 (20.4%) patients were positive for *S. aureus* (95 female, 119 male), of which 32 were MRSA isolates (14.95%). The MRSA rate in the whole cohort was 3.05%).

31 of the CHROMagar MRSA plates inoculated with the swabs used for the LightCycler® MRSA Advanced Test were positive for MRSA. Only 27 of the plates inoculated with the swabs used for the Detect-Ready® MRSA Kit were positive for MRSA. This difference is presumably due to improper sample collection technique leading to different amounts of material on the two heads of the swab. Only the samples tested positive for MRSA in the corresponding chromagar culture were included in the determination of true positive and false positive PCR results, except two cases with a positive MRSA result in both PCRs, but only on one of the culture plates. Those samples were defined as true positive results in the PCR as well, resulting in 32 culture positive samples. Of the 1049 samples, 21 samples could not be analyzed with the Detect-Ready® MRSA Kit, including two of the positive samples, due to a limited number of test kits.

### PCR results

The LightCycler® MRSA Advanced test detected MRSA in 42 samples, 27 of these samples were also positive on the CHROMagar, resulting in 27 true positive and 15 false positive results. Of the 1005 samples tested as MRSA negative in the LightCycler® MRSA Advanced test, 5 samples were positive on the CHROMagar and classified as false negative results (Table 1).

**Table 1:** Comparison of PCR results.

	LightCycler MRSA Advanced (Roche)	Detect-Ready MRSA (MDI)
**total no. of samples**	1049	1028

culture results BBL CHROMagar MRSA II				
**positive after 24 h**	29	2.76%	24	2.33%
**positive after 48 h**	31	2.96%	25	2.43%
**negative**	1018	97.04%	1003	97.57%

PCR results				
**positive**	42	4.00%	19	1.81%
**negative**	1005	95.81%	989	94.28%
**invalid**	2	0.19%	20	1.91%

result definition				
**true positive**	27	2.57%	15	1.46%
**false positive**	15	1.43%	4	0.39%
**false negative**	5	0.48%	11	1.07%
**true negative**	1000	95.33%	978	95.14%

test performance (binary classification)		
**clinical sensitivity**	84.38%	57.69%
**specificity**	98.52%	99.59%
**positive predictive value**	64.29%	78.95%
**negative predictive value**	99.50%	98.89%

The table lists the results of the MRSA screening with the LightCycler MRSA Advanced and the Detect-Ready MRSA test. True and false positive and negative values were determined by comparing the PCR results to the corresponding culture result. The results of the CHROMagar MRSA plates were set as gold standard. All positive cultural results were confirmed by the microbiological laboratory of our clinic. Two samples were tested positive in both PCRs and on one of the cultures and were treated as positive cultural samples for the determination of true und false positive values as well. Negative results of the Detect-Ready MRSA test are results not identified as MRSA by the test software.

With the Detect-Ready® MRSA Kit only 1028 samples could be processed. The test identified 19 samples as MRSA positive, 15 of those samples were positive on CHROMagar and therefore classified as true positive, the other four samples were classified as false positive. The other 989 samples were tested as MRSA negative; in detail 173 were identified as MSSA, 183 as a mixture of MSSA and coagulase negative staphylococci (CoNS), 324 as CoNS, and 309 as negative (Table 2). Ten of the MSSA and CoNS positive and one of the CoNS positive samples showed MRSA growth on CHROMagar and were classified as false negative in the PCR (Table 1).

**Table 2:** Results of the Detect-Ready MRSA PCR.

	no.	%
**samples**	1028	-
**MRSA**	19	1,85
**MSSA**	173	16,83
**MSSA+CoNS**	183	17,80
**CoNS**	324	31,52
**NEG**	309	30,06
**INV**	20	1,95

The lists the itemized results of the Detect-Ready MRSA assay. Additionally to the detection of MRSA the Detect-Ready MRSA PCR is able to differentiate the MRSA negative results into MSSA, MSSA+CoNS, CoNS, and negative results. MRSA: Methicillin-resistant *Staphylococcus aureus*; MSSA: Methicillin-sensitive *Staphylococcus aureus*; CoNS: Coagulase-negative *Staphylococcus aureus*; NEG: negative; INV: invalid.

Overall 22 specimens lead to invalid results upon testing with the LightCycler® MRSA Advanced Test (2/1049) and the Detect-Ready® MRSA Test (20/1028). All specimens were negative on CHROMagar MRSA and were not included in the calculation of clinical sensitivity, specificity, PPV, and NPV of the respective test.

The LightCycler® MRSA Advanced Test achieved a better clinical sensitivity than the Detect-Ready® MRSA Kit (84.38% versus 57.69%) in our clinical setting. The low sensitivity of the Detect-Ready® MRSA Kit was basically due to a number of samples falsely identified as a combination of MSSA and CoNS. The negative predictive values were nonetheless high (>98.5%) for both tests. The specificity and the positive predictive value were higher for the Detect-Ready® MRSA Kit (99.59% and 78.95% versus 98.52% and 64.29% in the LightCycler® MRSA Advanced Test) (Table 1).

Samples with MRSA growth on CHROMagar and negative PCR results were reanalyzed. The cultured MRSA isolates retrieved from the CHROMagar plates were used as templates for the respective PCR. In this approach three of five isolates from samples with false negative results in the LightCycler® Advanced MRSA-PCR were tested positive, one negative. One cultured sample was missing and could not be reanalyzed. The isolate tested negative was MRSA positive in the than performed Detect-Ready® MRSA Test. Concerning the Detect-Ready® MRSA PCR, the analysis of the isolates from 11 false negative PCR samples resulted in eight MRSA positive and three negative PCRs (one negative, one CoNS, one MSSA+CoNS). All three negative samples were tested positive in the LightCycler® MRSA Advanced Test. The four samples that failed to be detected in one of the PCR assays were sent to Alere Technologies GmbH for genotyping. All four MRSA strains belonged to the ST5/ST225-MRSA-II strain. SCC*mec* typing revealed that all had the SCC*mec*-cassette type II.

## Discussion

MRSA is still a growing problem in health care settings leading to increased costs and patient risks. Identification of colonized patients is the first step in the containment of MRSA spreading. Different diagnostic tests are available for the identification of colonized patients so far.

Factors to consider for the choice of a MRSA screening platform include sensitivities, specificities, turnaround time, costs, and ease of interpretation, which had been shown to vary considerably [35]. MRSA screening methodology is already widely in use even though accuracy values of the respective tests are not all sufficiently tested in a clinical routine setting or even in comparison to already better evaluated tests.

Here, we compared the Detect-Ready® MRSA Kit (MDI) with the methods used routinely for MRSA screening in our hospital (LightCycler® MRSA Advanced Test (Roche) and CHROMagar MRSA (BD)).

Costs and turnaround time play an important role in the decision which assay is the test of choice. Reagent and instrument costs are much higher and turnaround times shorter for the PCR assays. LightCycler® MRSA Advanced Test had the shortest turnaround time with less than 2 hours. Processing of the Detect-Ready MRSA Test was finished within 5 hours requiring a considerable hand-on time. Results from cultures were available after 24 hours of incubation at the earliest (94.78% of MRSA positive cultures could be identified after 24 hours (Table 1)). Interpretation of positive results was easy to perform for both PCR platforms, with software programs providing clearly arranged result lists after every PCR run

Both test methods are based on the detection of the SCC*mec*-*orfX* junction. The staphylococcal cassette chromosome *mec* (SCC*mec*) is the carrier of the resistance-gene *mecA*. It integrates into the *S. aureus* genome in the region of the open reading frame *orfX* which is specific for *S. aureus* [21]. Therefore, an amplification product is obtained in SCC*mec* carrying *S. aureus* but not in SCC*mec* carrying CoNS. SCC*mec* PCRs have been reported to have high sensitivities and specificities and are used in several commercial available MRSA detection kits [22], [23], [24], [25], [27].

In this study both tests had high specificities (LightCycler® MRSA Advanced Test: 98.52%, Detect-Ready® MRSA Kit: 99.59%). LightCycler® MRSA Advanced Test was more sensitive for the detection of MRSA (84.38%) than Detect-Ready® MRSA Kit, but both tests had poorer sensitivities in our real-life study setting than reported in previous studies [36]. Especially the Detect-Ready® MRSA Kit had a surprisingly low clinical sensitivity (57.69%). This might be due to a software/cut off problem of the Detect-Ready MRSA test, as the software calculates automatically if MRSA or a mixed population of staphylococci is present in the samples (10 of the culture positive samples were identified as MSSA and CoNS and one as CoNS). After re-analysis of the false negative results using the cultured strains as PCR template, three of the five false negative LightCycler® Advanced MRSA Test results and eight of the 11 false negative Detect-Ready® MRSA results tested positive, which would increase the sensitivities so 93.75% and 88.46%, respectively, if the samples were tested positive in the first PCR.

While the Detect-Ready® MRSA PCR detected more false negative results, the LightCycler® MRSA PCR produced significantly more false positive results (LightCycler® MRSA: 15 FP, Detect-Ready® MRSA: 4 FP, p<0.05) resulting in a lower predictive positive value (64.29% versus 78.95%).

The detection of false positive and false negative results could have had several reasons. Recent studies reported a *S. aureus* strain (LGA251) resistant to methicillin but negative for *mecA* [28], [29]. This strain harbours a divergent *mecA* homologue with a different organization than other SCC elements leading to a false negative result. Another study from Denmark [30] revealed that a specific common SCC*mec* clone was frequently not detected in a commercial MRSA assay leading to the conclusion that local diversities play an important role in the performance of MRSA assays, as undetectable low prevalence strains could become widespread among *S. aureus*. In our study, most of the samples which were false negative in the PCR proved to be positive in a second PCR approach using the isolated MRSA cultures. In those cases most probably an inoculum effect (plates were inoculated prior to MRSA PCRs), low concentration on the epithelium of MRSA, or inhibitory PCR effects seem to be the cause for retrieving false negative results in the PCR assays. Four samples were tested negative again in the second PCR approach, but all samples were MRSA positive in the conquering assay. Genotyping revealed that all strains belonged to the ST5/ST225-MRSA strain with a SCC*mec*-cassette type II, a common type in this region. Future sequence analysis of these four strains would be interesting to determine why they could not be detected by the PCR assays. Minor changes in the sequence of the primer binding sites could be the reason. Maybe there is a MRSA subpopulation in our region which is not detectable with certain commercial assays comparable to the MRSA clone in the above mentioned Danish study [30].

The reason for false positive PCR results can be *orfX* genes in CoNS homologue to the *S. aureus* variant or SCC*mec* cassettes lacking *mecA* [31], [23], [32], [33]. To avoid false positive results in this case, the Detect-Ready® MRSA Test detects a second *S. aureus* specific marker (*nuc*) and the *mecA* gene additionally to the SCC*mec-orfX* amplicon. Overall, this approach enables the differentiation between MRSA and MSSA in addition. This may be important in surgical patients, as preoperative detection of MSSA nasal carriage and decolonization of the patient could reduces the risk of surgical site infections due to *S. aureus* [34]. 95% of the cultured MSSA were detected by the Detect-Ready MRSA Assay. The assay detected MSSA in nearly twice the number of samples than the culture method. This may either be due to false positive PCRs, or to a lack of selectivity of the here uses culture method for *S. aureus*. Columbia agar enables the growth of numerous bacteria which can overgrow small amounts of MSSA which are then not detected. A comparison of a selective MSSA agar with the Detect-Ready Assay would be an interesting approach for the future but could not be conducted in scope of this study.

In this study PCR based methods were compared to the direct plating on chromogenic agar. Since not performed under routine conditions neither, no broth-enriched culture was used in the study. Thus, our study is limited by the possibility that broth-enrichement would have lead to more culture positive results. PCR results defined here as false positive could also reflect a detection of non viable MRSA. The reason for choosing the routine culture method as standard was our demand to focus on patients colonised with a considerable amount of still viable MRSA, which determines its transmissibility.

Our study is further limited by the fact, that the false positive samples (amplicons) could not be characterized by molecular methods to examine whether other genetic abnormalities lead to a positive PCR result. No additional data concerning former antibiotic treatment was retrieved for the patients, so it could not be excluded that the detection of false positive samples was due to prior antibiotic treatment, nor to what extent. In contrast to our results, Peterson et al [26] showed, a higher sensitivity of 95% of the the LightCycler® MRSA Advanced Test in comparison to direct culture, possibly due to an exclusion of patients with antibiotic therapy active against MRSA.

In conclusion, our data show that the LightCycler® MRSA Advanced Test demonstrated a better clinical sensitivity compared to the Detect-Ready® MRSA Kit. We would recommend additional cultural testing in a clinical setting to close the diagnostic gap and to avoid false results. With the CHROMagar MRSA the majority of the positive results (95%) were achieved already after 24 hours, demonstrating this culture based test as a relatively fast, cheep and reliable screening method in situations where no immediate results are needed.,.
